# Supplementary material for: A Cluster Analysis of the Acceptance of a Contact Tracing App—The Identification of Profiles for the Italian Immuni Contact Tracing App
Source: Healthcare (Basel). 2022 May 11;10(5):888. doi: 10.3390/healthcare10050888 (PMC9140954; doi:10.3390/healthcare10050888)
Supplement: Supplementary file 1 [file healthcare-10-00888-s001.zip › healthcare-1695432-supplementary.pdf]

Dati Socio-Anagrafici e variabili categoriali (Socio-Demographic Data and categorical variables)

Genere

Età (in anni compiuti)

Livello di studio

Regione dove hai trascorso la quarantena

Quante App hai installato sul tuo smartphone? (0-5, 6-10, più di 10)

Ti sei mai rifiutato di installare una App a causa dei permessi richiesti? (SI/NO)

Conosci la App Immuni? (SI/NO)

Hai già scaricato la App Immuni? (SI/NO)

Quale tipo di dato sul tuo smartphone consideri più privato (Dati del profilo, Dati sulla mia condizione di salute, Le mie foto, La mia posizione e i miei percorsi, Altro)

Utilità Percepita (Perceived Usefulness) - Fortemente in disaccordo 1 - Fortemente d'accordo 5

Utilizzare l'app IMMUNI sarà utile per gestire la mia salute

Utilizzare l'app immuni sarà vantaggioso per gestire meglio la mia salute

Utilizzare l'app immuni sarà utile per me

Utilizzare l'app IMMUNI sarà prezioso per la mia salute

Facilità d'Uso Percepita (Perceived Ease of Use) - Fortemente in disaccordo 1 - Fortemente d'accordo 5

Imparare ad utilizzare l'app immuni per avere informazioni riguardanti la salute sarà facile per me

Le mie interazioni con l'app IMMUNI saranno chiare e comprensibili

Ritengo che l'app IMMUNI sia uno strumento flessibile

Sarà facile per me diventare abile nell' utilizzare l'app IMMUNI

Influenza sociale (Social Influence) - Fortemente in disaccordo 1 - Fortemente d'accordo 5

Userò la App Immuni se i miei familiari mi chiederanno di farlo

Userò la App Immuni se anche i miei familiari la useranno

Userò la App Immuni se chi mi sta vicino la userà

Le persone a me vicine approverebbero l'utilizzo della App Immuni

I miei amici approverebbero l'utilizzo della App Immuni

Il mio medico di base approverebbe l'utilizzo della App Immuni

Le persone che hanno più influenza su di me ritengono che dovrei imparare ad usare strumenti per gestire la mia salute come la App Immuni

Le persone per me più importanti ritengono che dovrei usare strumenti per gestire la mia salute come la App Immuni

Condizioni facilitanti (Facilitating Conditions) - Fortemente in disaccordo 1 - Fortemente d'accordo 5

Ho i prerequisiti tecnici necessari per utilizzare la App Immuni

Possiedo il know-how tecnico (ovvero le conoscenze e le abilità operative) per utilizzare la App Immuni

Se mi venissero date le risorse, le opportunità e le informazioni necessarie per usare la App Immuni, sarebbe più facile utilizzare questo strumento

Attitudine (Attitude) - Fortemente in disaccordo 1 - Fortemente d'accordo 5

Utilizzare l'app immuni sarebbe una buona idea

Utilizzare l'app immuni sarebbe un'idea saggia

Mi piace l'idea di utilizzare l'app immuni per gestire la mia salute in relazione al covid

Utilizzare l'app immuni sarebbe un'esperienza piacevole

Intenzione (Behavioral intention) - Fortemente in disaccordo 1 - Fortemente d'accordo 5

Supponendo di avere accesso all'app Immuni, avrei intenzione di usarla

Supponendo di avere accesso all'app Immuni, prevedo che la userei

Ho pianificato di usare l'app Immuni nei prossimi _3_ mesi

Autoefficacia nell'uso del computer (Computer Self-Efficacy) - Per niente fiducioso 1 - Totalmente fiducioso 10

"Riuscirei a utilizzare l'app Immuni se…"

Non ci fosse nessuno in giro che mi dicesse cosa fare

Non avessi mai usato una tecnologia simile prima

Avessi solo i manuali di istruzioni come riferimento

Avessi visto qualcun altro usarla prima di provarla io stesso

Potessi chiamare qualcuno per chiedere aiuto se mi bloccassi

Qualcuno mi avesse aiutato ad iniziare

Avessi avuto molto tempo per completare le attività per le quali la tecnologia è stata fornita

Avessi solo la possibilità di usare l'help dell'applicazione

Qualcuno mi avesse mostrato prima come si fa

Avessi usato una tecnologia simile prima di questa per risolvere la stessa problematica

Gravità percepita verso le malattie croniche (Perceived severity to chronic diseases) - Fortemente in disaccordo 1 - Fortemente d'accordo 5

Ho paura di affrontare le complicanze eventualmente derivanti da COVID-19

Se dovessi affrontare le complicanze derivanti dal COVID-19 potrei avere difficoltà con la mia vita lavorativa (o domestica)

Se dovessi affrontare le complicanze derivanti da COVID-19 ciò potrebbe ostacolare le mie relazioni personali

Se dovessi affrontare le complicanze derivanti da COVID-19 potrei essere perseguitato a lungo dai problemi che ne deriveranno

Coscienziosità verso la salute (Health consciousness) - Fortemente in disaccordo 1 - Fortemente d'accordo 5

Ho l'impressione di aver sacrificato molto per la mia salute

Mi considero molto attento alla salute

Penso di prendere molto in considerazione la salute nella mia vita

Penso che sia importante conoscere bene come stare in salute

La mia salute è molto preziosa per me, e sono pronto/a a sacrificare molte cose per essa.

Ho l'impressione che le altre persone facciano più attenzione di me alla loro salute (R)

Non mi chiedo di continuo se qualcosa sia buono per me (R)

Non credo proprio di pensare spesso se tutto quello che faccio sia salutare (R)

Non voglio chiedere in ogni momento a me stesso se le cose che mangio sono giuste per me (R)

Mi soffermo spesso sulla mia salute

Sono pronto a fare quel che serve per avere una buona salute

Disimpegno morale (Moral Disengagement) - Completamente in disaccordo 1 - Completamente d'accordo 7

Giustificazione morale (Moral Justification)

Non usare la App Immuni è necessario per garantire il proprio diritto alla privacy

Ritengo ingiusto pretendere che i cittadini accettino di condividere le proprie informazioni personali

E' immorale che la App Immuni consenta di raccogliere cosi' tante informazioni sulla persona

Etichettature eufemistica (Euphemistic labelling)

Credo che non ci sia proprio niente di male nel non usare la App Immuni

La App Immuni e' l'ennesima trovata che non risolve nulla

Dietro all' App Immuni ci sono sicuramente interessi economici

Confronto vantaggioso (Advantageous Comparison)

Pensando a come i politici pensano ai propri interessi personali, non credo proprio che non usare la App Immuni faccia tutta questa differenza

Non usare la App Immuni consente di boicottare il progetto di guadagno di chi ci sta dietro

Meglio il rischio COVID che il rischio di perdita della Liberta'

Distorsione delle conseguenze (Distorsion of Consequences)

Farsi tracciare con la app Immuni non ha alcun effetto positivo sulla lotta al coronavirus

La App immuni potrebbe creare molti problemi sociali

La scelta di non adottare la App immuni e' giustificata dalla volontà' di evitare ritorsioni personali

Diffusione di responsabilità (Diffusion of Responsibility)

Nessuna delle persone che io conosco sembra disposta a rinunciare alla propria privacy non vedo perché dovrei farlo io usando la App Immuni

Se le persone decidono di non usare la App Immuni e' perche' tanto si sa che la maggioranza non lo fara' e non servira' a niente

Scegliere di condividere le proprie informazioni quando gli altri non lo fanno non ha alcun senso

Attribuzione di colpa (Attribution of Blame or Guilt)

Boicottare la App Immuni farebbe capire al governo quanto ha sbagliato

Se nessuno adotterà la App Immuni sara' perche non c'e' stata giusta e corretta informazione

L'insuccesso della App Immuni sarà' dovuto all'incapacità' di chi ci governa

Fiducia nelle Istituzioni (Istitutional Trust) - Per niente 1 - Moltissimo 7

Quanto ti fidi…

del Governo

del Presidente del Consiglio

dell'Istituto Superiore di Sanità

del Presidente della tua Regione

del Commissario Straordinario della Protezione Civile

Orientamento politico (Political Orientation)

Nella seguente scala come ti collochi <u>politicamente</u>: Molto di sinistra 1 - Molto di destra 7

Valori politici fondamentali (Core Political Values) - Completamente in disaccordo 1 - Completamente d'accordo 7

Legge e ordine (Law and Order)

È giusto per il nostro Paese adottare delle misure restrittive sulle libertà civili per garantire la sicurezza dei cittadini.

L'ordine deve essere preservato ad ogni costo, anche se ciò potrebbe limitare le libertà civili.

Sarebbe una buona idea limitare alcune libertà individuali se così facendo si potesse garantire più ordine.

Libertà civili (Civil Liberties)

È estremamente importante rispettare la libertà degli individui di essere e fare ciò che vogliono.

La cosa più importante per la nostra società è la difesa delle libertà civili.

Il diritto alla libertà individuale non deve essere violato e deve essere mantenuto a tutti i costi.

Privacy - Fortemente in disaccordo 1 - Fortemente d'accordo 5

Mi fa sentire a disagio che l'uso di Immuni possa essere facilmente monitorato.

Mi infastidisce che le informazioni legate all'uso di Immuni possano essere

utilizzate anche tra anni.

Sento che l'uso di Immuni rende più facile invadere la mia privacy.

Responsabilità prosociale (Prosocial responsibility) - Completamente in disaccordo 1 - Completamente d'accordo 7

Tutti noi, cittadini italiani, dovremmo fare la nostra parte per impedire che le persone nel nostro paese vengano infettate dal virus.

Sono anche io come cittadino singolo responsabile di progettere gli altri cittadini dalla pandemia.

Mi sento personalmente responsabile a contribuire alla salute delle persone nel nostro paese nell'attuale pandemia di coronavirus.

Luogo percepito di causalità (Perceived Locus of Causality) - Completamente in disaccordo 1 - Completamente d'accordo 7

Penso di usare la app Immuni perché:

rispecchia i miei valori

è importante per me a titolo personale

mi sentirei in colpa se non lo facessi

non voglio essere criticato perchè non lo faccio

capisco perchè è importante farlo

mi sento in qualche modo obbligato a farlo

lo trovo sensato

gli altri disaproverebbero se non lo facessi
